# Remedy for Dental Neuralgia

**Published:** 1859-07

**Authors:** 


*Remedy for Dental Neuralgia.*—M. Balloy recommends the following mixture for the relief of this painful affliction: Acetate of Morphia, a grain and a half, Acetic Acid, two drops, Cologne water, two fluid drachms. He soaks cotton with it and introduces it into the ear of the effected side.

